# Bioinformatical analysis of the key differentially expressed genes for screening potential biomarkers in Wilms tumor

**DOI:** 10.1038/s41598-023-42730-w

**Published:** 2023-09-16

**Authors:** Linghao Cai, Bo Shi, Kun Zhu, Xiaohui Zhong, Dengming Lai, Jinhu Wang, Jinfa Tou

**Affiliations:** 1 Department of Neonatal Surgery, Children’s Hospital, Zhejiang University School of Medicine, Nation Clinical Research Center for Child Health, Zhejiang Provincial Clinical Research Center for Child Health, Hangzhou, China; 2 Department of Pathology, Children’s Hospital, Zhejiang University School of Medicine, Nation Clinical Research Center for Child Health, Zhejiang Provincial Clinical Research Center for Child Health, Hangzhou, China; 3 Department of Thoracic and Cardiovascular Surgery, Children’s Hospital, Zhejiang University School of Medicine, Nation Clinical Research Center for Child Health, Zhejiang Provincial Clinical Research Center for Child Health, Hangzhou, China; 4 Department of Oncology Surgery, Children’s Hospital, Zhejiang University School of Medicine, Nation Clinical Research Center for Child Health, Zhejiang Provincial Clinical Research Center for Child Health, Hangzhou, China

**Keywords:** Tumour biomarkers, Paediatric cancer

## Abstract

Wilms tumor (WT) is the most common pediatric renal malignant tumor in the world. Overall, the prognosis of Wilms tumor is very good. However, the prognosis of patients with anaplastic tumor histology or disease relapse is still poor, and their recurrence rate, metastasis rate and mortality are significantly increased compared with others. Currently, the combination of histopathological examination and molecular biology is essential to predict prognosis and guide the treatment. However, the molecular mechanism has not been well studied. Genetic profiling may be helpful in some way. Hence, we sought to identify novel promising biomarkers of WT by integrating bioinformatics analysis and to identify genes associated with the pathogenesis of WT. In the presented study, the NCBI Gene Expression Omnibus was used to download two datasets of gene expression profiles related to WT patients for the purpose of detecting overlapped differentially expressed genes (DEGs). The DEGs were then uploaded to DAVID database for enrichment analysis. In addition, the functional interactions between proteins were evaluated by simulating the protein–protein interaction (PPI) network of DEGs. The impact of selected hub genes on survival in WT patients was analyzed by using the online tool R2: Genomics Analysis and Visualization Platform. The correlation between gene expression and the degree of immune infiltration was assessed by the Estimation of Stromal and Immune cells in Malignant Tumor tissues using the Expression (ESTIMATE) algorithm and the single sample GSEA. Top 12 genes were identified for further study after constructing a PPI network and screening hub gene modules. Kinesin family member 2C (*KIF2C*) was identified as the most significant gene predicting the overall survival of WT patients. The expression of *KIF2C* in WT was further verified by quantitative real-time polymerase chain reaction and immunohistochemistry. Furthermore, we found that *KIF2C* was significantly correlated with immune cell infiltration in WT. Our present study demonstrated that altered expression of *KIF2C* may be involved in WT and serve as a potential prognostic biomarker for WT patients.

## Introduction

Wilms tumor (WT), also known as nephroblastoma, is the most common pediatric renal malignant tumor, accounting for 90% of pediatric renal tumors and 8% of all pediatric cancers^1,2^. More than 14,000 children worldwide are diagnosed with WT each year, and 5,000 of them die from WT, with regional variations in mortality rates^3^. The prognosis is poor for patients who have anaplastic histology or favorable histology with disease relapse. It is estimated that 15% of patients with WT will relapse, and the long-term survival rate for those with relapsed WT is only 50%^4^. In recent years, the standard treatment for WT has been nephrectomy and combined chemotherapy, either alone or in combination with radiotherapy, depending on tumor histology and disease stage. The combination of histopathological examination and molecular biology to predict prognosis and guide treatment in patients with WT is the most common^5^. In recent years, many biomarkers have been identified to have a significant impact on the prognosis of WT. Patients with diffuse anaplasia and TP53 alterations tend to have poor prognosis^6,7^. Chromosomal alterations such as the loss of heterozygosity (LOH) for 1p and 16p, gain of 1q are often associated with an inferior prognosis^8^. Some novel candidate biomarkers such as Prohibitin (*PHB*), *SIX1/2*, microRNA have also been found to serve significant roles in WT^9–11^. However, a small number of children still died from recurrence, metastasis, resistance to chemotherapy, and other complications^4,12^. There is still a need to develop effective and feasible methods for predicting possible outcomes for WT patient subsets who currently have less favorable outcomes. Numerous studies have demonstrated that the tumour immune microenvironment (TIM) plays a significant role in cancer progression and immunotherapy outcome^13^. It has been found that tumor-infiltrating immune cells have prognostic value in a variety of tumor types, and that they are associated with the expression of certain biomarkers^14^. Identifying biomarkers associated with WT may have important and urgent implications for early prediction and personalized treatment of WT.

Currently, the microarray technique has become increasingly popular in life science research purposes. Data generated by high-throughput techniques such as RNA sequencing and microarrays can be used to investigate the molecular mechanisms that drive tumor progression. These studies can provide useful insights and serve as a basis for further investigation^11,15^. The Gene Expression Omnibus (GEO) is a database conducted by the National Center for Biotechnology Information (NCBI). By screening differentially expressed genes (DEGs), it can be possible to investigate molecular signals and correlations, as well as analyze gene regulatory networks by analyzing gene expression profiles in various tumor types.^16^. Currently, several studies have demonstrated that genes are crucial for the incidence and development of WT^2,17,18^.

Previous studies have shown that kinesin family member 2C (*KIF2C*) plays a critical role in microtubule depolymerization, bipolar spindle formation, and chromosome segregation, all of which are involved in mitosis and the cell cycle^19–21^. A higher level of microtubule KIF2C protein increased chromosome instability^22–24^. In addition, *KIF2C* has been identified in cytoskeletal remodeling during tumor metastasis^25^. There has been extensive study of the involvement of *KIF2C* in cancer progression and development, including studies of non-small cell lung cancer, endometrial carcinoma, glioma, and hepatocellular carcinoma^26–28^. However, the expression pattern of *KIF2C* in WT remains uninvestigated.

Our study aimed to identify and validate potential novel biomarkers associated with WT by integrating two microarray datasets from the GEO database. *KIF2C* was screened as an oncoprotein in WT occurrence and development. We found that *KIF2C* is negatively correlated with immune cell infiltration. Meanwhile, we performed polymerase chain reaction (qRT-PCR) and immunohistochemical (IHC) analyses to validate the expression of *KIF2C* in WT tissues.

## Materials and methods

### Identification of differentially expressed genes

In this study, we searched the GEO database and selected two gene expression profiles (GSE66405 and GSE11024)^29–31^. These GSE datasets were downloaded from the NCBI database and all GSE datasets consisted of two groups: WT and normal tissue. The GSE66405 datasets contained 28 WT samples and 4 non-cancerous samples and the GSE11024 datasets contained 26 WT samples and 4 non-cancerous samples. Processing and normalizing these data can be accomplished using a variety of methods. Here, we used an online tool called GEO2R, which can effectively detect differentially expressed mRNAs between WT samples and non-cancerous samples. The tool is capable of analyzing almost any GEO profile so that two groups can be compared under the same experimental conditions^32^. DEGs were screened with the adjusted *P* value of < 0.05 and |log fold change| of > 1.5.

### Functional pathway enrichment analysis of differentially expressed genes

Pathways associated with identified DEGs were enriched by Gene Ontology (GO) and Kyoto Encyclopedia of Genes and Genomes (KEGG), using the Database for Annotation, Visualization and Integrated Discovery (DAVID) database^33^. A GO term enrichment analysis was conducted to explore the biological significance of DEGs. *P* < 0.05 was considered significant.

### Protein–protein interaction network visualization

To further understand the related function interactions among proteins, DEGs were used to construct a simulated PPI network. A protein–protein interaction (PPI) network was visualized using the Search Tool for Retrieval of Interacting Genes (STRING) database and Cytoscape to evaluate the functional interactions between proteins^34,35^. Following that, we selected appropriate modules of PPI network using Molecular Complex Detection (MCODE)^36^. There was a cutoff point of 2 for the degree and 0.2 for the node score, a k-score of 2, and a maximum depth of 100. The cytoHubba plug-in was used to identify hub genes^37^. The top 12 genes were screened out with degree ≧ 10 as hub genes.

### Survival analysis of differentially expressed genes

An online tool R2: Genomics Analysis and Visualization Platform can be used to assess the effect of selected individual genes on the survival of WT patients, in which you will find survival information for various cancer patients^38^. The WT patients were divided into two groups (high versus low) based on the hub gene expression. The overall survival (OS) of WT patients was obtained and plotted on R2.

### Ethics statement

Written informed consents were obtained from the parents for all cases. This study has been reviewed and approved by the Ethics Committee of the Children's Hospital of Zhejiang University School of Medicine (2020-IRB-049-A1). All methods were carried out in accordance with relevant guidelines and regulations.

### qRT-PCR experiment

qRT-PCR was used to measure *KIF2C* mRNA expression. Total RNA was extracted from 3 randomly selected WT tissues and 3 adjacent WT tissues with Tissue Total RNA kit (5100050, Simgen, China) according to the protocol. Subsequently, reverse transcription was then performed on the extracted RNA to produce cDNA. Real-time PCR was performed using a StepOne Plus Real-Time PCR System, using an Applied Biosystems Prime Script ^tm^ RT Master Mix, containing 20 ng cDNA and 10 μL of each primer. Cycling conditions were 1 cycle at 95 °C for 30 s, 40 cycles at 95 °C for 5 s, and 60 °C for 34 s. PCR amplification specificity was determined by melting curve analysis for each reaction. The expression level of *KIF2C* was normalized to GAPDH and calculated using 2^−∆∆Ct^ method. The primer sequences for *KIF2C* were forward (GAAGAGAGCTCAGGAGTATG) and reverse (TTCTGTGCTCTTCGATAGGA). The GAPDH primer sequences were forward (GGGGCTCTCCAGAACATC) and reverse (TGACACGTTGGCAGTGG).

### Immunohistochemical staining

Seventeen samples for IHC were collected from WT tissues removed from post-operative patients in the Department of Surgical Oncology. Three of the samples contained both tumor tissue and adjacent normal tissue. Paraffin-embedded histological slices were cut into 5 μm slices after 10% formalin fixation. Sections were stained with hematoxylin and eosin (H&E) or deparaffinized with dimethylbenzene and dehydrated through 100, 95, 85, and 75% ethanol. The antigen retrieval procedure was performed in 0.01 mol/L sodium citrate buffer by heating in a pressure cooker for 10 min and cooling naturally. Endogenous peroxidase was blocked by incubation with 3% H_2_O_2_ for 15 min at room temperature. The sections were subsequently washed in triethanolamine buffered saline with Tween (TBST) solution, blocked with bovine serum albumin (BSA) for 20 min and incubated with anti-KIF2C (28372-1-AP, 1:200, Proteintech, China) overnight at 4 ℃. Following three TBST solution washes, the sections were incubated with HRP-conjugated secondary antibody for 20 min at room temperature. All slides were counterstained with diaminobenzidine (DAB) solution (Sangon Biotech, Shanghai, China). Finally, sections were counterstained with hematoxylin, dehydrated, cleared and mounted. The expression level of KIF2C was determined by immunohistochemical score (IHS). IHS were determined based on staining intensity (SI) and percentage of immunoreactive cells (PR). Staining intensity (SI): 0: no neoplastic cells showed membranous-like staining; 1: ≤ 10% of neoplastic cells showed incomplete, weak circumferential membranous-like staining; 2: ≤ 10% of neoplastic cells showed complete, weak circumferential membranous-like staining. 3: > 10% of neoplastic cells showed incomplete circumferential membranous-like staining, 4: > 10% of neoplastic cells showed complete circumferential membranous-like staining; Neoplastic cells with different staining intensities were assigned different scores, and the sum was calculated.

$$IHS = SI_{1} \times PR_{1} \% + SI_{2} \times PR_{2} \% + SI_{3} \times PR_{3} \% + SI_{4} \times PR_{4} \%$$

### Immune infiltration analysis

Estimation of Stromal and Immune cells in Malignant Tumor Tissues using the Expression date (ESTIMATE) algorithm was used to calculate ESTIMATE score, Immune score and Stromal score by the ESTIMATE 1.0.13 R package. Based on these scores, we assessed the difference between tumor microenvironment exhibited by high and low *KIF2C* expression groups. Furthermore, the microenvironment cell population counter (MCP-counter) 1.2.0 R package was used to assess the composition of immune cells in the high and low expression groups of *KIF2C*. A further analysis of the enrichment scores of 28 immune-related gene sets was carried out by using ssGSEA in the high and low *KIF2C* expression groups.

### Statistical analysis

All data were analyzed using GraphPad Prism 8.0 statistical software and R programming language (version 4.0.2). Differences between groups were compared using t-test. Statistical significance was determined by a *P* value of < 0.05.

### Ethics approval and consent to participate

Informed consent was obtained for tissue samples used for qRT-PCR and IHC. This study has been reviewed and approved by the Ethics Committee of the Children's Hospital of Zhejiang University School of Medicine (2020-IRB-049-A1). All methods were carried out in accordance with relevant guidelines and regulations.

### Consent for publication

Written informed consent was obtained from all legally authorized open-source data for the publication of identifiable data.

## Result

### Screening of differentially expressed genes

**Figure 1:**
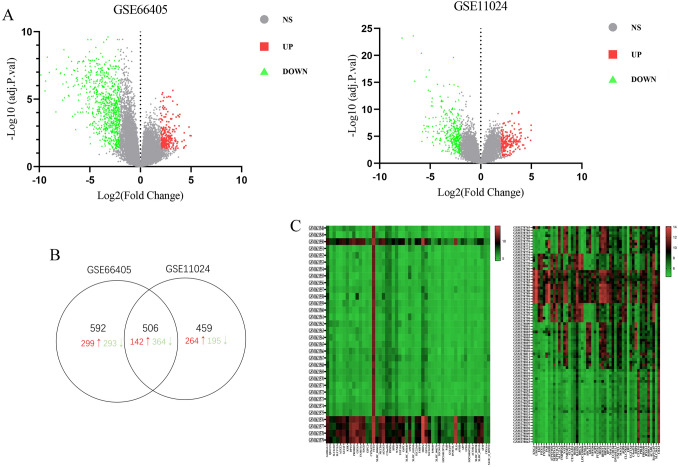
The identification of DEGs compared between WT and normal kidney tissue in gene expression profiling datasets. (**A**) Volcano plots of differential gene expression. Red indicates up-regulated DEGs; green indicates down-regulated DEGs. (**B**) Venn plot for DEGs that were shared between the datasets. (**C**) Hierarchical cluster analysis (heat maps) of the top 50 up-regulated and down-regulated common DEGs of the two datasets. *DEGs* differentially expressed genes.

A total of 1598 and 965 DEGs compared between WT and normal kidney tissue were identified from the GSE66405 and GSE11024 datasets, respectively. Based on the two sets of sample data, the differential expression of multiple genes across the two microarrays is shown as volcano plots (Fig. 1A). In total, 506 genes between the two datasets were shown to be identical based on bioinformatics analysis (Fig. 1B). Compared with normal samples, 142 genes were up-regulated and 364 genes were down-regulated in WT samples (Table 1). Heat maps of the top 50 genes up-regulated and down-regulated expression are shown in Fig. 1C.

**Table 1:** List of consistent DEGs in two datasets, including 142 up-regulated and 364 down-regulated DEGs. DEGs, differentially expressed genes.

Regulation	DEGs
Up-regulated (n = 142)	*WASF1, CXCR4, TPX2, MLF1IP, LRRN1, LRFN5, LMNB2, ANLN, BIRC5, PLEKHO1, DUSP4, ROR2, CEP170, C5orf13, TUBB, RGS4, PCDH18, KDELC1, TCF4, KIF14, C1orf112, SFRP2, MEIS1, TUBA1A, COL5A2, SIX2, KIF4A, HAS2, TYMS, MELK, NDC80, RNF175, OIP5, E2F7, PSRC1, PTTG1, CDCA5, VASH2, CENPE, LMNB1, CRABP2, TACC3, CPXM1, MEX3A, FSCN1, CCNB2, PRC1, CEP55, ELOVL2, ZNF423, MFAP2, FBXL7, RAD51AP1, VCAN, CYP27C1, MKI67, PTGFRN, MARCKSL1, NMU, BOC, CENPK, TLE4, MLLT11, KIAA0101, GINS2, MYB, MEX3B, UNC5B, GPX7, BASP1, TMEM45A, FBN2, CACNB3, CHST1, EZH2, FZD2, LRRC17, CENPH, NNAT, TRO, CDH2, ENAH, GPR64, CDCA7L, EYA1, MTHFD2, NPTX2, FZD7, PCDHB2, KIF2C, FBXO5, CENPA, CDCA7, CDC20, BUB1, TRIP13, ZNF521, COL13A1, CDC7, LYPD1, MARCKS, SACS, IGF2, ATAD2, UBE2C, MND1, POLE2, CKAP2, COL2A1, PCDHB10, HK2, MDK, TOP2A, FANCI, KIF15, BUB1B, OLFML2B, DTL, MAGEL2, ETV5, CCND2, RGMA, KIF20A, ENC1, SOX11, SIX1, COL5A1, LAMA4, MAP4K4, PRAME, CTHRC1, CHN1, RBP1, TTK, CDKN3, SCG5, NCAPG, CENPM, PDGFC, CENPF, NUSAP1, CCDC88A*
Down-regulated (n = 364)	*RALYL, BBOX1, CUBN, TMEM139, TMC4, GDF15, HMGCS2, FAM134B, RGN, GDA, NOSTRIN, GOT1, SLC13A1, SUSD2, SLC22A2, HSD11B2, GPC5, A1CF, BHMT, MAOA, GJB1, F2RL1, SLC16A5, KL, SLC22A6, GPD1, C1orf168, CRHBP, ASS1, AQP2, SLC17A3, TSPAN7, MIOX, ACADL, FGFR3, AGT, SULT1C2, VTCN1, TFAP2B, CLEC3B, NQO2, HLF, SLC39A5, C12orf59, SMTNL2, AKR7A3, SLC3A1, PBLD, PERP, ATP1A1, MUC15, UNC5CL, MT1G, FXYD2, PPARGC1A, CYP3A5, ALDH6A1, EGF, RCAN1, NTN4, HSPA2, TYRP1, C1orf115, TMEM176A, ATP6V1A, WNK4, SGK2, IMPA2, NPY1R, CLDN10, SOST, LRRC19, ACE2, SCNN1B, ECHDC3, PODXL, MUC20, RAMP3, NPNT, GAL3ST1, RAB25, C5orf32, MME, PHYH, GGT6, AQP6, SLC23A3, GPR160, CLCNKB, APOM, CA12, RHBG, SLC22A5, GRAMD1C, LGALS2, NR3C2, EMCN, BLNK, PDZK1IP1, GGT1, ALDH1L1, SLC23A1, CGNL1, ASL, GATA3, GIPC2, SLC12A3, SLC44A3, MT1E, ACY1, GADD45A, OLFM4, THSD7A, KIF12, SLC22A7, NDRG1, AQP3, NPHS2, UPB1, NAPSA, ERP27, ENPEP, PVALB, CBX7, FOXI1, CPEB4, ACSL1, SLC16A4, SLC5A10, S100A2, DMRT2, GLDC, FBP1, SPTBN2, GALNT14, GPR56, GLRX, KNG1, KRT7, DPEP1, PLG, SLC7A7, ARG2, GLS, HAO2, EPS8L2, MT1F, HNF1B, TGFBR3, UMOD, DIO1, SLC6A12, SOSTDC1, CRYL1, PPP1R1A, SLC22A12, NUAK2, SLC27A2, G6PC, TMED6, CEACAM1, SCNN1G, SLC13A3, CYB5A, NCOA7, KCNJ16, LEPREL1, UGT8, CYFIP2, SLC4A4, AOX1, SERPINA5, HRSP12, EHHADH, PIPOX, LNX1, GULP1, TMEM37, HSPA12A, HYAL1, SLC44A4, RBP5, PTGER3, COBLL1, SPINK1, HPN, CXCL14, SLC17A1, EHF, FRMD3, CAPN3, NAT8, FOXQ1, SERPINA6, GK, CALB1, GPR116, DEFB1, SLC22A18, ACAT1, FMO4, TMEM30B, ANGPTL3, ATP1B1, TFCP2L1, DDAH1, ALDH4A1, DNASE1L3, CRYAB, SCIN, MUC1, EFHD1, ELF3, RAP1GAP, C9orf66, CLDN2, PIGR, ATP6V1G3, FUT6, FOLR1, RCAN2, MAL, SLC47A1, IRF6, COL4A3, CYP4F3, KHK, ESRRG, CLDN7, ABCB1, ELOVL7, SLC34A1, SERINC2, C14orf105, FAM149A, SLC22A8, C4orf19, FABP3, SLC22A11, TUFT1, BHMT2, SLC16A9, WWC1, PLS1, ITGB6, SDC4, ASPA, PCK1, SLC26A4, IRX1, DPYS, TSPAN33, NOX4, GABARAPL1, TINAG, IL17RB, GABRA2, CLMN, KCNJ10, UGT1A6, TMEM27, FXYD4, S100A6, RHCG, CYP4A11, AGXT2, GRB14, MT1X, MST1, FMO1, SHMT1, TMPRSS2, RAB17, EPHX2, CLDN19, ATP6V0A4, GLYATL1, ACSS1, HPD, PDE2A, PLCG2, KCNJ1, MAOB, ACADSB, RBP4, RASGRP1, FABP1, CDH16, COL4A4, CLCN5, FGF9, PLCL1, SDC1, SLC1A1, CDH1, PD, ALDH8A1, SLC7A9, AZGP1, PRODH, C10orf116, CX3CL1, EHD3, WDR72, RBM47, ALDH1A1, RAPGEF3, PAQR5, IRX2, CA2, CRYZ, MFSD4, TMEM116, RASD1, GLYAT, DDC, ANPEP, GSTM3, SLC47A2, NR1H4, PTPRO, AQP1, CLYBL, MSRA, TSPAN1, KIF13B, PCCA, RNF186, ABP1, ACMSD, PLLP, COBL, ATP6V0D2, AP1M2, SCNN1A, PDZK1, ENPP6, GBA3, CLDN8, GPX3, KLK1, CGN, TCEA3, CLRN3, ARSE, MT1H, PRSS8, ALDOB, FTCD, AGMAT, KCNJ15, GATM, CISH, OGDHL, ATP6V1B1, SLCO4C1, DUSP9, VAV3, USH1C, PRODH2, C11orf54, PEPD, TOB1, PAH*

### GO and KEGG pathway enrichment analysis

**Figure 2:**
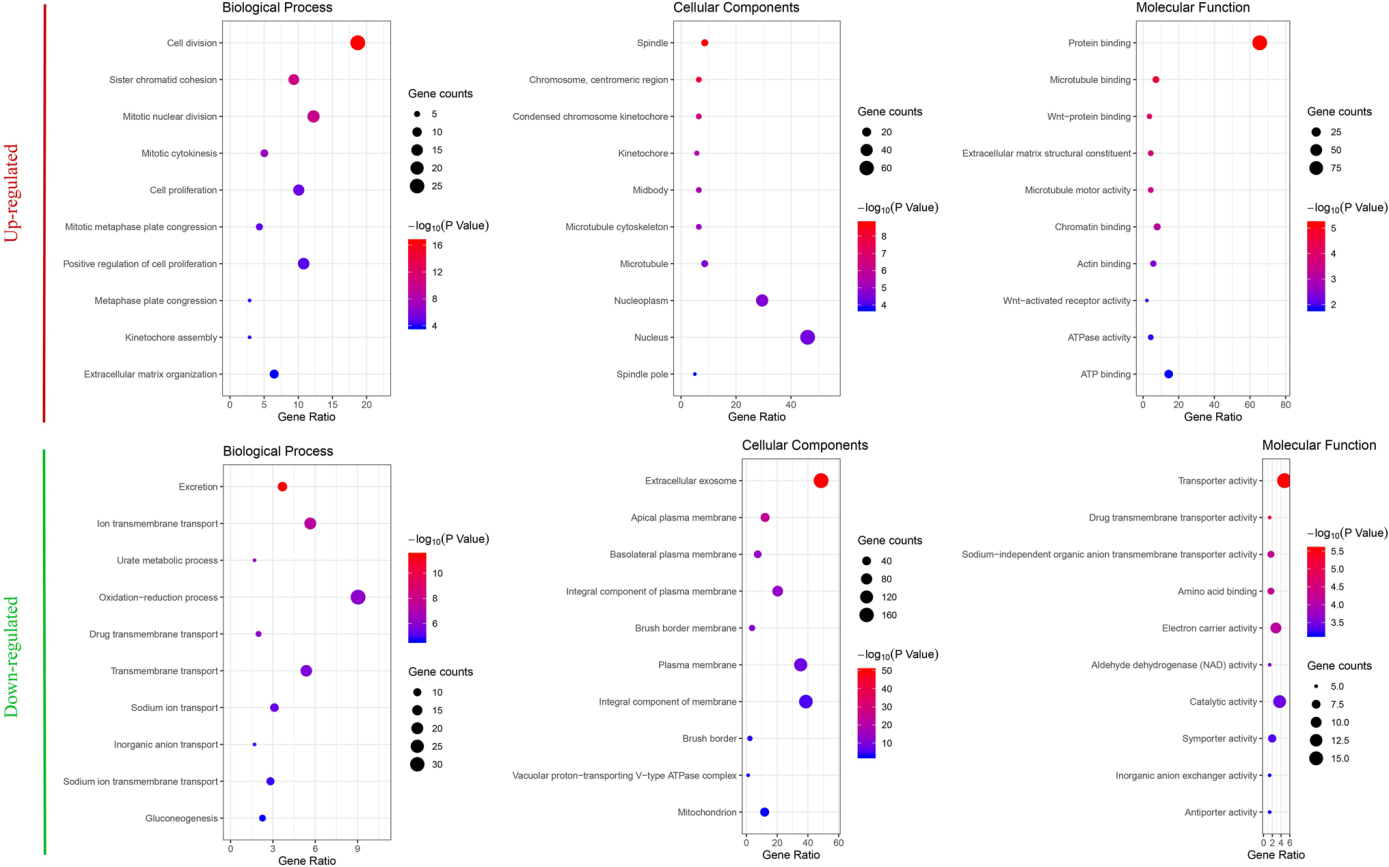
Top 10 functional analyses of the overlapping up-regulated and down-regulated DEGs according to biological process, cellular components and molecular function. *DEGs* differentially expressed genes.

**Figure 3:**
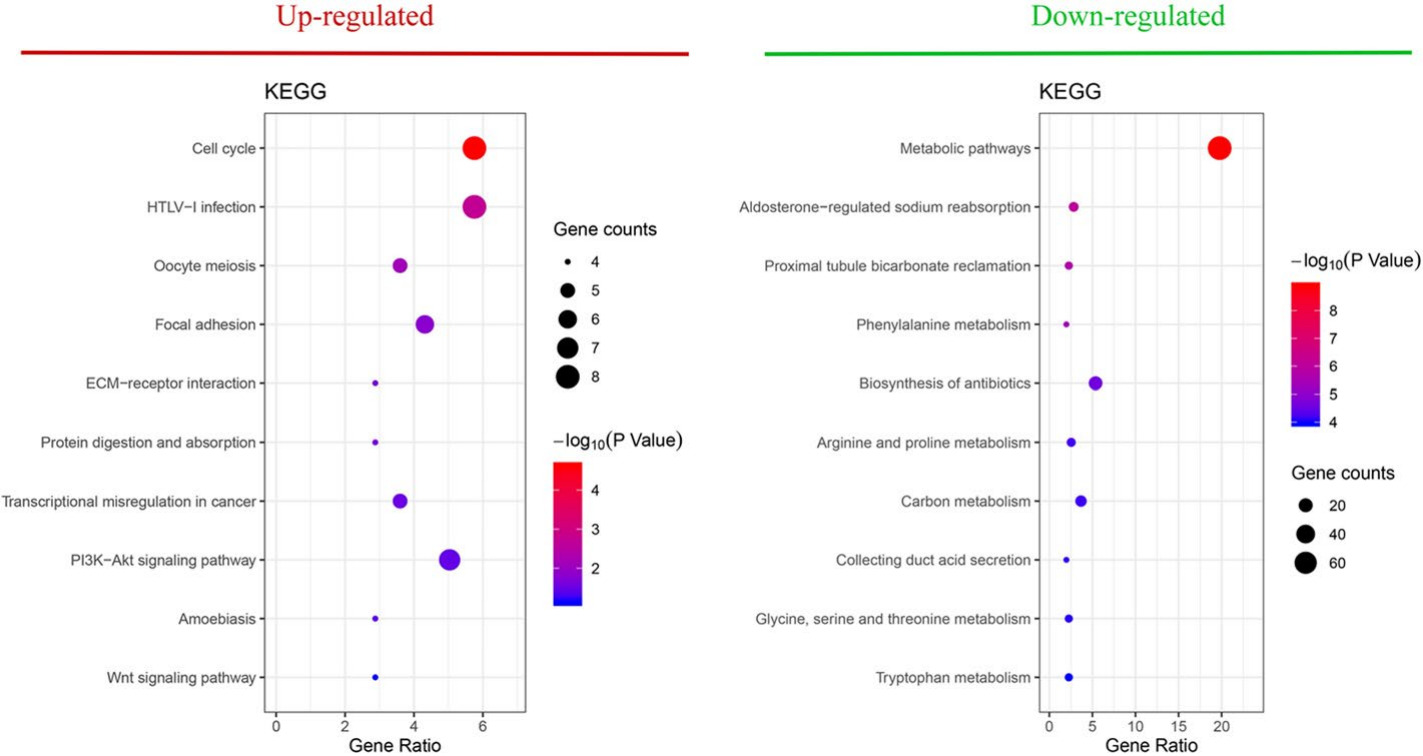
Top 10 KEGG pathway enrichment analyses of the overlapping up-regulated and down-regulated DEGs. *KEGG* Kyoto Encyclopedia of Genes and Genomes, *DEGs* differentially expressed genes.

To further understand the diversity of functions performed by DEGs, we conducted GO functions and KEGG pathway enrichment analyses for DEGs on the DAVID platform. By extending analysis of the enriched biological process (BP), cellular components (CC) and molecular function (MF), we gained a deeper understanding of the biological functions of overlapping DEGs. According to *P* values, the ggplot2 R package was used to construct the bubble map of the top 10 biological processes (Fig. 2). The up-regulated genes were mainly related to the cell division, sister chromatid cohesion, mitotic nuclear division in enriched GO terms for BP; meanwhile nucleus, spindle, and chromosome centromeric region for CC; and protein binding and microtubule binding for MF. The down-regulated genes were mostly related to excretion, long transmembrane transport in enriched GO terms for BP; extracellular exosome for CC; and transporter activity and drug transmembrane transporter activity for MF (Fig. 2). Furthermore, in the KEGG pathway enrichment analysis, there were four main pathways that were associated with up-regulated DEGs, including the cell cycle, HTLV-I infection, oocyte meiosis, and focal adhesion, while almost only one metabolic pathway was enriched in the down-regulated genes (Fig. 3).

### PPI network construction and Hub gene selection

**Figure 4:**
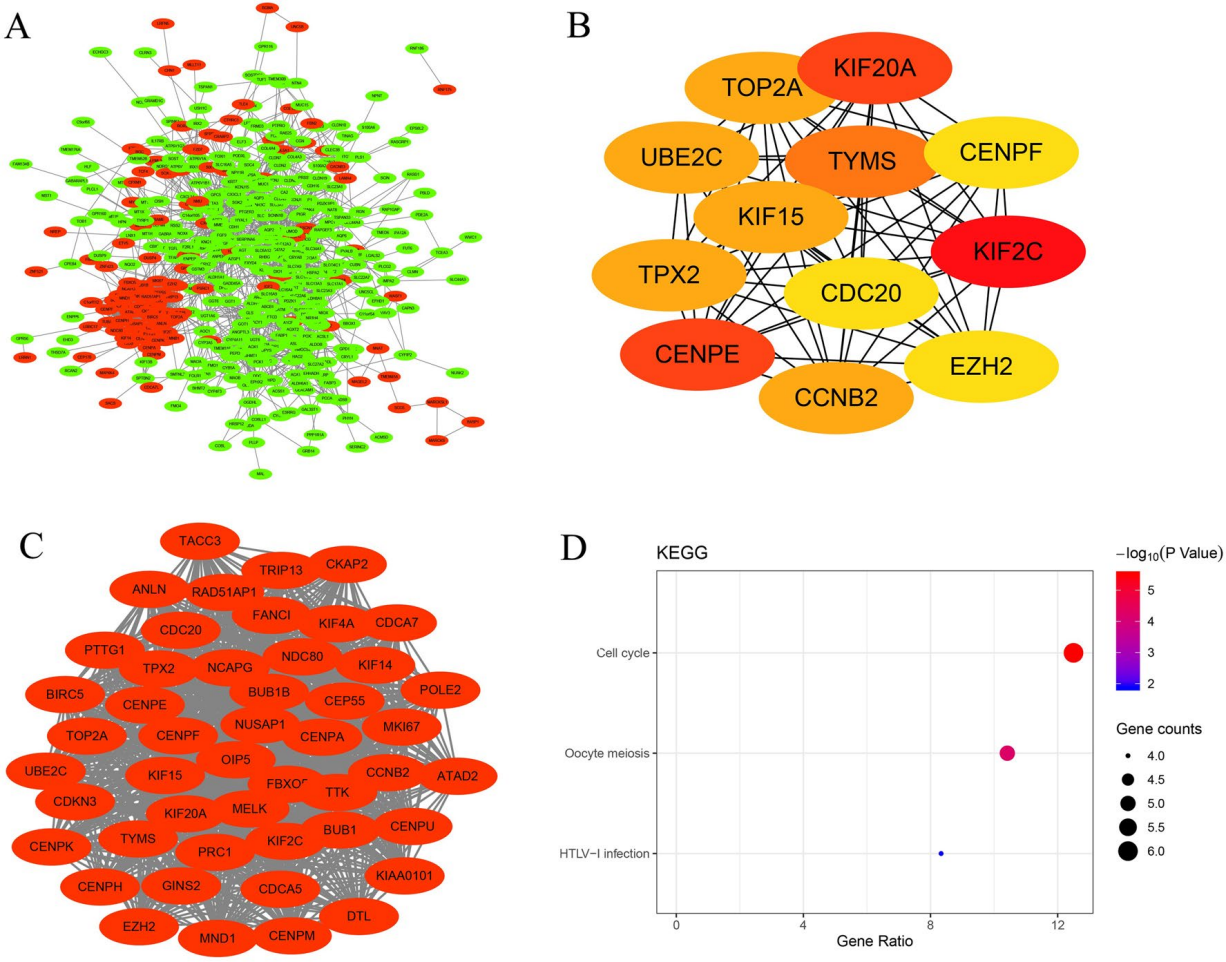
Construction of protein–protein interaction networks of DEGs by STRING. (**A**) Differential gene protein interaction network. Red indicates up-regulated DEGs; Green indicates down-regulated DEGs. (**B**) Top 12 hub genes with a higher degree of connectivity. (**C**) Top module screened from the PPI network by MCODE plug-in. (**D**) KEGG pathway enrichment analysis of the top module. *DEGs* differentially expressed genes, *STRING* Search Tool for Retrieval of Interacting Genes, *KEGG* Kyoto Encyclopedia of Genes and Genomes, *MCODE* Molecular Complex Detection.

The PPI network was constructed by selecting 458 nodes and 2638 edges consisting of 142 up-regulated genes and 364 down-regulated genes (Fig. 4A). The top 12 hub genes including *KIF2C*, centromere protein E (*CENPE*), kinesin family member 20A (*KIF20A*), thymidylate synthetase (*TYMS*), topoisomerase II alpha (*TOP2A*), kinesin family member 15 (*KIF15*), ubiquitin-conjugating enzyme E2C (*UBE2C*), cyclin B2 (*CCNB2*), TPX2 Microtubule Nucleation Factor (*TPX2*), enhancer of zeste 2 polycomb repressive complex 2 subunit (*EZH2*), cell division cycle 20 (*CDC20*), and centromere protein F (*CENPF*) were identified by the cytoHubba plug-in (Fig. 4B). All the 12 hub genes identified in the PPI network were up-regulated in WT compared to normal kidney samples. The MCODE plug-in was used to determine the most significant module from the PPI network based on the degree of importance, which included 48 nodes and 1048 edges (Fig. 4C). An analysis of gene enrichment revealed that genes in this module are mainly involved in the cell cycle, oocyte meiosis, and HTLV-1 infection (Fig. 4D).

### Kaplan–Meier survival plotter

**Figure 5:**
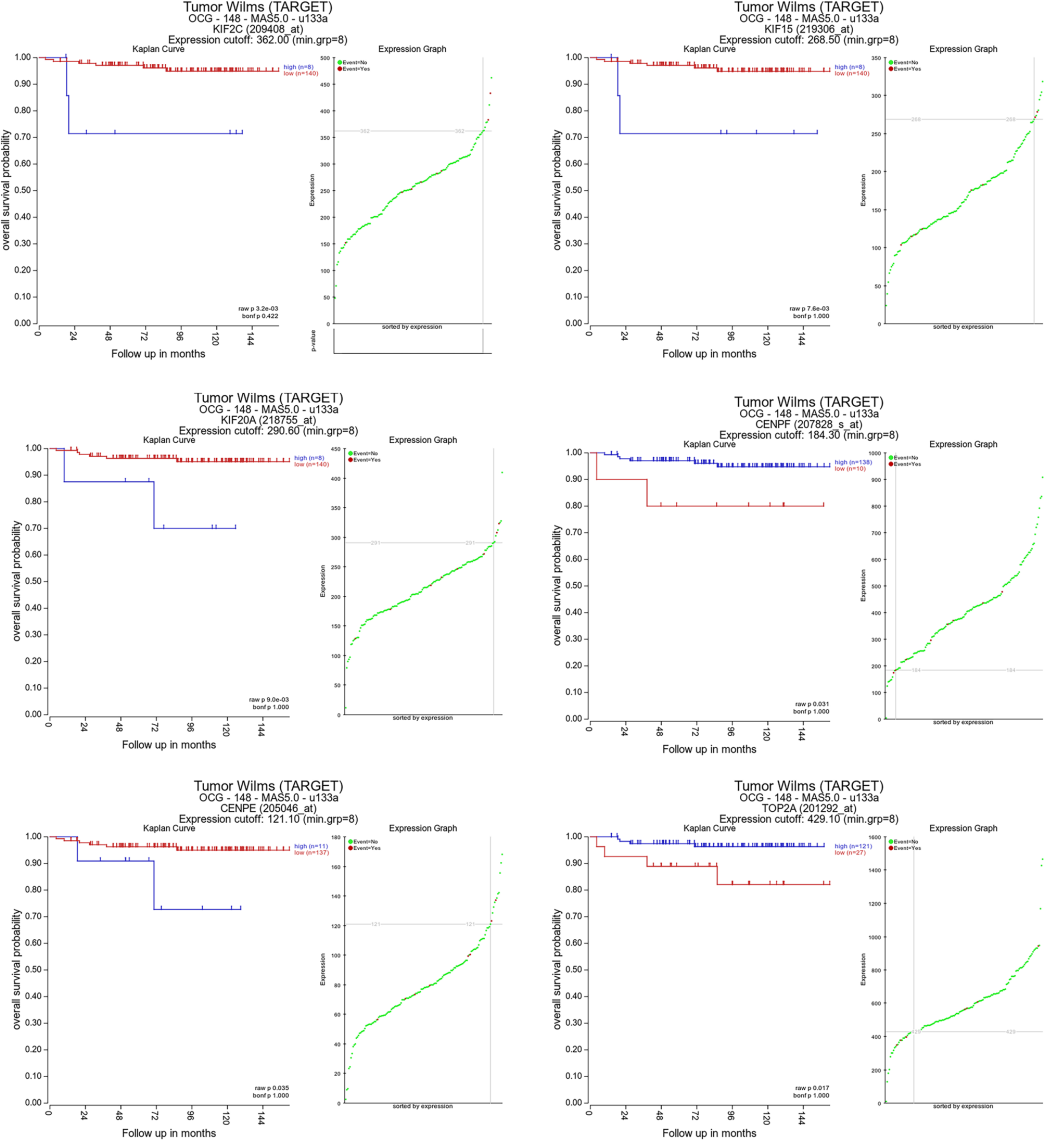
The relationship between the most significant 6 hub genes expression and the overall survival of patients with Wilms tumor. *KIF2C*, kinesin family member 2C; *KIF15*, kinesin family member 15; *KIF20A*, kinesin family member 20A; *CENPF*, centromere protein F; *CENPE*, centromere protein E; *TOP2A*, topoisomerase II alpha.

The prognostic information related to the 12 hub genes can be found on the platform R2: Genomics Analysis and Visualization. High expression level of *KIF2C* (*p* = 3.2e-03), *KIF15* (*p* = 7.6e-03), *KIF20A* (*p* = 9.0e-03), *CENPF* (*p* = 0.031) and *CENPE* (*p* = 0.035) was found to be associated with worse OS of WT patient (Fig. 5). High expression level of *TOP2A* (*p* = 0.017) was related to better performance at patients' overall survival. Among these genes, *KIF2C* played the most important role in prognosis. Thus, *KIF2C* was chosen for further analysis.

### qRT-PCR to verify the expression of KIF2C

**Figure 6:**
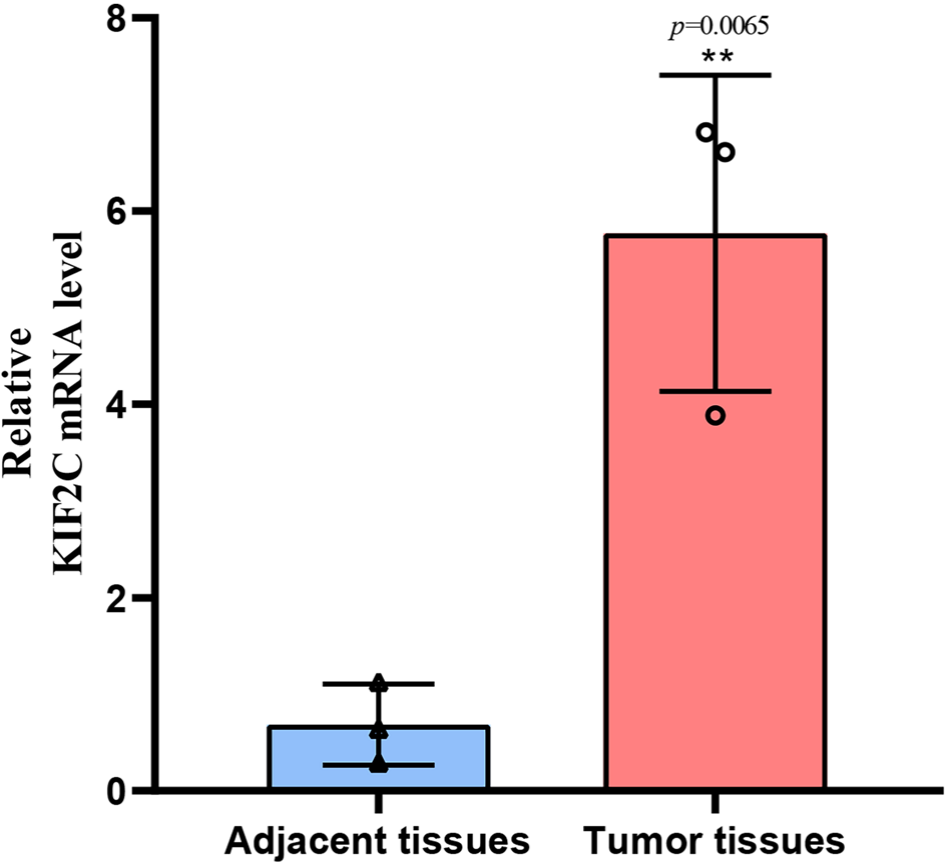
The *KIF2C* mRNA level was up-regulated in WT tissues by quantitative real-time polymerase chain reaction.

The expression of *KIF2C* transcriptional mRNA level was verified in tissues using qRT-PCR in 3 available WT patient samples. Compared with 3 adjacent WT normal tissues, there was a significant increase in the expression level of *KIF2C* in WT tissues (*p* = 0.0065) (Fig. 6).

### Immunohistochemistry to verify the expression of KIF2C

**Figure 7:**
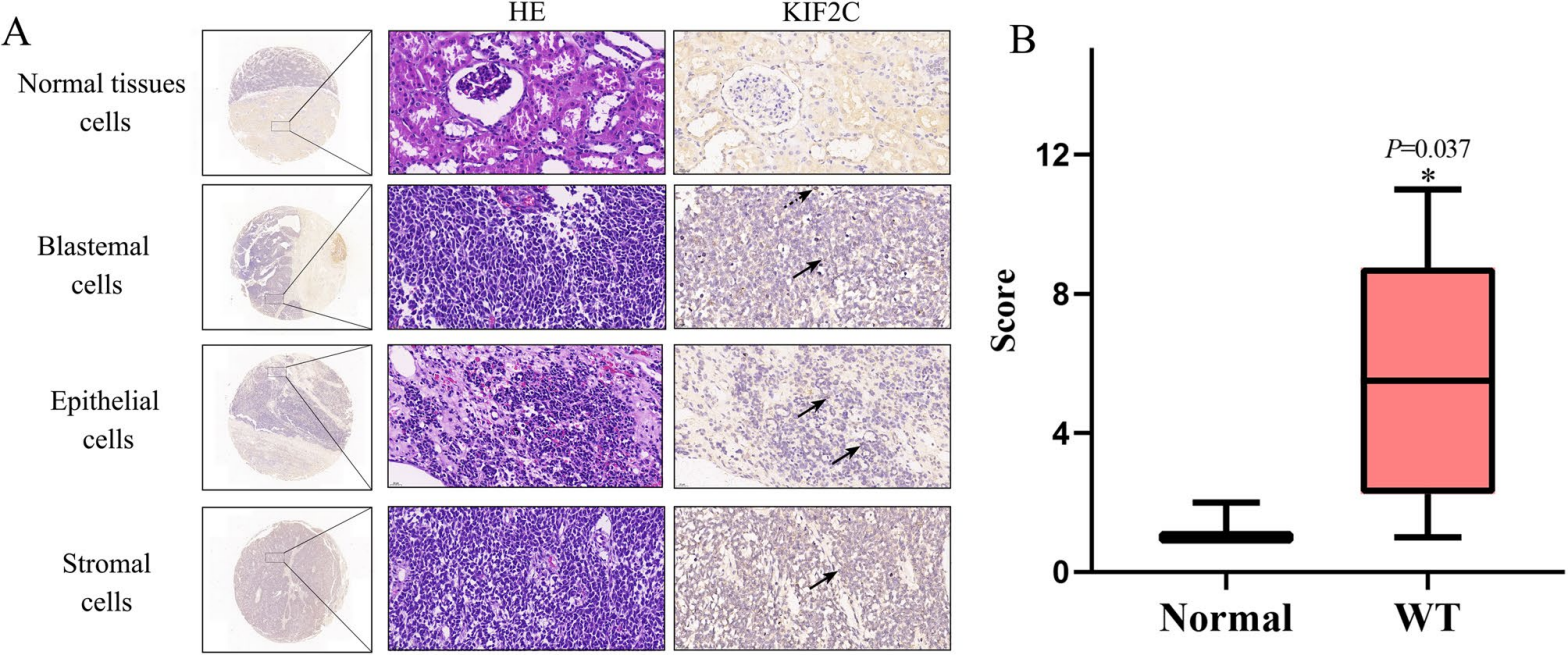
Upregulation of KIF2C expression in WT. (**A**) Representative immunohistochemical staining results showed the protein level expression of KIF2C in in blastemal, epithelial and stromal cells of WT tissues as well as in normal kidney tissue cells. (**B**) Expression scores between WT and normal tissues. ↑, neoplastic cells showed incomplete, weak circumferential membranous-like staining. ⇡, neoplastic cells showed complete circumferential membranous-like staining. *WT* Wilms tumor, *HE* hematoxylin–eosin staining, *KIF2C* Kinesin family member 2C.

Immunohistochemical staining demonstrated the expression of KIF2C in blastemal, epithelial and stromal cells of WT tissues as well as in normal kidney cells. Compared with normal kidney tissues, the positive immunohistochemistry staining of WT tissues was mainly manifested as membranous-like staining, and most of neoplastic cells showed incomplete, weak circumferential membranous-like staining (Fig. 7A). The expression socre of KIF2C in WT tissues was significantly higher (*p* = 0.037) than that of adjacent normal tissues (Fig. 7B).

### The correlaion between *KIF2C* expression and immune infiltration

**Figure 8:**
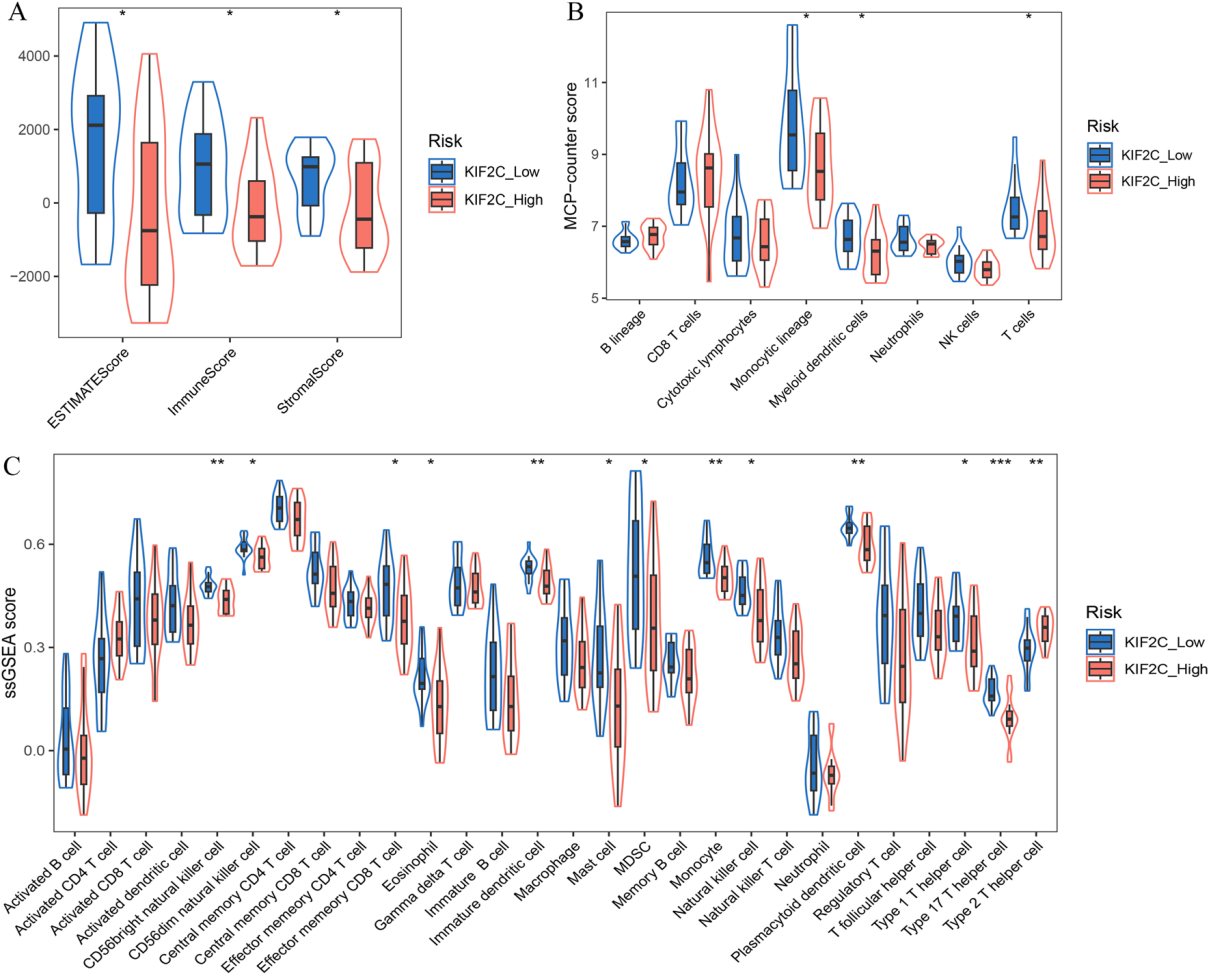
Correlation between *KIF2C* expression and immune cell infiltration in Wilms tumor. (**A**) ESTIMATE scores, Immune scores and Stromal scores between high *KIF2C* group and low *KIF2C* group, respectively. (**B, C**) The abundance of various cells in the tumor microenvironment analyzed by MCP-counter algorithm and ssGSEA. *P < 0.05, **P < 0.01, ***P < 0.001.

In order to assess the relationship between *KIF2C* expression levels and tumor microenvironment, we first compared the differences in ESTIMATE, Immune and Stromal score among the high and low *KIF2C* expression groups. As compared to the low expression group, the high *KIF2C* expression group's ESTIMATE score, immune score and stromal score were all significantly lower (Fig. 8A). Furthermore, the MCP-counter algorithm and ssGSEA analyses were performed identify the difference in infiltrating immune cells in the tumor microenvironment of WT patients. The results of MCP-counter showed a decreased number of tumor-infiltrating immune cells, including monocytic lineage, myeloid dendritic cells and T cells in the high *KIF2C* expression group compared with the low expression group (Fig. 8B). Consistently, ssGSEA results also showed that the vast majority of infiltrating immune cells were fewer in the high-expression group than low-expression groups, except that Type 2T helper cell were more in the high-expression group (Fig. 8C).

## Discussion

Despite advances in the surgery and treatment of WT, the prognosis of patients with anaplastic tumor histology or disease relapse is still poor. Therefore, it is important to find reliable tumor biomarkers and investigate possible molecular mechanisms to understand the pathogenesis and abnormal biological behavior of WT, as well as to identify novel therapeutic targets of WT. Although numerous studies have demonstrated that genes contribute significantly to WT development and incidence, most of them were limited to cohort studies^39,40^. In this study, we identified genes with significant differences in two GEO databases (GSE66405 and GSE11024 datasets), and finally found 506 common altered DEGs through bioinformatic method, including 142 up-regulated genes and 364 down-regulated genes. The DAVID database was used to analyze GO terms and KEGG pathways for these 506 DEGs. To screen out the hub genes closely related to WT, a PPI network was constructed. The top 12 hub genes with higher degree of connectivity were filtered from the PPI network. The gene hub genes were primarily associated with the cell cycle, meiosis of the oocyte, and HTLV-1 infection according to functional and KEGG pathway enrichment analysis. We plotted survival curves for each of these hub genes using the Kaplan–Meier method and found that *KIF2C* had the most predictive value for WT survival. Finally, we verified the expression of KIF2C in WT tissues by qRT-PCR and IHC respectively. It is worth noting that the IHC results showed an expression of KI2C primarily in circumferential membranous-like areas. Most neoplastic cells showed weak incomplete membranous-like staining, and some neoplastic cells showed complete membranous-like staining. It is consistent with the results of the bioinformatic analysis we conducted. According to our analysis of GSE66405 and GSE11024, the log fold change values for *KIF2C* are 2.5 and 1.907768, respectively. This suggests that the expression of KIF2C in WT is different but not very strong compared with normal kidney tissues. Therefore, we used a scoring standard similar to that used for HER2 in breast cancer when assessing IHC results^41,42^. Our results show that the expression score of KIF2C in WT tissues was significantly higher (*p* = 0.037) than that of adjacent normal tissues. The expression scores of blastemal cells appear to be higher, which may be associated with a poor prognosis^21^.

A major finding of our study concerned the *KIF2C* gene. Kinesins are the microtubule-associated motors that produce mechanical work associated with adenosine-triphosphate (ATP) hydrolysis^43^. Kinesin family-13 is the critical regulator of microtubule dynamics during mitosis^44^. *KIF2C*, a mitotic centromere-associated kinesin, is the most representative member of the Kinesin family‐13, which is located in the cytoplasm throughout the cell cycle^45^. According to previous studies, *KIF2C* regulates mitosis and cell cycle by attaching microtubules to kinetochores, assembling spindles, and regulating chromosome congression^20–22,46^. In addition, *KIF2C* participates in cytoskeleton remodeling during tumor metastasis^47^. It has been reported that *KIF2C* can stimulate the proliferation and migration of gastric cancer cells as well as non-small cell lung cancer cells^25,48^. It would therefore be worthwhile to further research the role of *KIF2C* in WT.

The function of *KIF2C* is critical for the assembly of the spindle to repair microtubules and chromosomal abnormalities. Therefore, abnormal expression of *KIF2C* could theoretically contribute to the development of cancer by causing mitotic anomalies, chromosomal instability, and uncontrolled transcription^49^. A number of studies have demonstrated abnormal expression of *KIF2C* in cancers such as breast cancer, oral tongue cancer, hepatocellular carcinoma, and non-small cell lung cancer.^22,26,50,51^. In lung adenocarcinoma, Bai et al*.* demonstrated that *KIF2C* expression is closely associated with the relapse of the cancer and the stage of the tumor.^49^. Nakamura et al*.* identified that *KIF2C* gene expression in gastric cancer tissues (65 cases) was significantly higher than the expression in non-malignant tissues, and the higher expression of *KIF2C* was associated with lymphatic invasion (*p* = 0.01), lymph node metastasis (*p* = 0.04) and poorer survival rate (*p* = 0.008)^52^. Gnjatic et al*.* observed a significantly increased expression of *KIF2C* in malignant compared with autologous healthy colorectal tissues^53^. However, the *KIF2C* expression level in WT remains unclear. According to our study, bioinformatic analysis indicates that *KIF2C* expression is significantly higher in WT tissues. The results of qRT-PCR and IHC staining validated our bioinformatic analysis at mRNA and protein levels, respectively. These results, combined with our findings, suggest that *KIF2C* may act as a novel biomarker that may be associated with prognosis in patients with WT.

Recent studies have explored how the KIFs family regulates chromosome and spindle movements during mitosis and meiosis^54,55^. However, the mechanism of *KIF2C* dysregulation in WT remains largely unknown. It has been demonstrated that *KIF2C* seems to be related to a poor prognosis by regulating cell cycle signaling pathway. An et al. illustrated that *KIF2C* promotes endometrial cancer cell proliferation, migration, and invasion in vitro. In addition, they found that cells were prevented from entering the G2 phase when *KIF2C* expression was reduced, suggesting that *KIF2C* knockdown may induce cell cycle arrest by prolonging the G1 phases^27^. Li et al. found that down-regulation of *KIF2C* effectively inhibited the growth of liver cancer cells by decreasing the proliferation and increasing the G1 arrest^51^. Based on Gan et al.'s findings, *KIF2C* knockdown cells showed significantly reduced viability and colony formation^26^. It has therefore been suggested that *KIF2C* may contribute to the proliferation of malignancies. Additionally, the results of GO and KEGG analyses also revealed that some genes closely related to cell mitosis may play important roles in WT. The hub genes identified in this article were also found to be related to the regulation of cell cycle. The findings mentioned above further suggest that KIF2C may be involved in during the cell cycle in WT.

In mitosis, *KIF2C* regulates microtubule dynamics and ensures that chromosomes attach correctly to spindles^28^. Considering *KIF2C*'s role in regulating microtubule dynamics, it has the potential to be developed as a drug target. Microtubule-targeting agents disrupt microtubule dynamics, resulting in prolonged mitotic arrest and eventually cell death^56^. Hedrick et al*.* reported that using anti-microtubule drugs such as paclitaxel and vinblastine, the knockdown of *KIF2C* in HeLa cells significantly exacerbated the morphological defects of the microtubule cytoskeleton^57^. It has been hypothesized that *KIF2C* silencing synergizes and enhances the effects of paclitaxel on cancer cells by interfering with spindle separation, which prolongs mitotic time, resulting in mitosis and apoptotic disorders^58,59^. It is believed that blocking mitotic exit could be a promising anticancer strategy, particularly for treating cancers such as breast cancer that are resistant to chemotherapy^60,61^. Studies by Wei et al. demonstrated the role of *KIF2C* in the positive regulation of mTORC1 signaling in hepatocellular carcinoma, indicating that an mTOR inhibitor could be a potential therapeutic intervention in hepatocellular carcinoma^62^.

The infiltrating immune cells are a crucial aspect of the tumor microenvironment and have a profound impact on patient prognosis and the efficacy of immunotherapy^63^. It has been demonstrated by Tu et al*.* that increased expression of *KIF2C* correlates with altered levels of immune cell infiltration in gliomas^64^. In hepatocellular carcinoma tissues, Li et al*.* observed that all T cell infiltration level decreased significantly compared to non-tumour tissues, indicating that low levels of T cell infiltration may represent a poor clinical prognosis^27^. However, the relationship between *KIF2C* expression and immune infiltration in WT is unknown. In this study, our analysis suggests that *KIF2C* is significantly negatively correlated with most immune cells in WT. First, we found that the proportion of infiltrating immune cells, including monocytic lineage, myeloid dendritic cells and T cells, was reduced in the high *KIF2C* expression group compared with the low expression group. Secondly, the results of ssGSEA analysis showed that the vast majority of infiltrating immune cells were fewer in the high-expression group than low-expression groups. Those findings are in agreement with those of the published studies. Mardanpour et al*.*’s research has shown that patients with high CD8 + tumor-infiltrating lymphocyte levels are associated with favorable clinical outcomes^65^. Tian et al*.* found that riskscore and immune score were negatively correlated in WT patients. Their research suggested that impaired anti-tumor immunity may be responsible for the poor prognosis of WT patients at high risk. Research into the mechanisms that disrupt the anti-tumor immune response will contribute to the development of more effective treatments^66^.

Our study has some limitations. Firstly, the reason why multiple datasets were selected for integrated analysis in this research is to obtain DEGs with higher specificity. However, due to experimental variations between the different datasets, the analysis based on public datasets may lack some reliability. The experiment type of two datasets selected are both expression profiling by array. We used an online tool named GEO2R to process and normalize gene expression, so that the two datasets can be effectively compared under the same conditions. Although we have validated in PCR and IHC with a small number of tissue samples, there is still a lack of relevant cell experiments, large clinical sample experiments and reliable corresponding clinical data. It is important to note that although our research identified some mechanisms that may contribute to WT genesis and prognosis, they must be further investigated in cell experiments. In brief, we utilized bioinformatics methods to integrate two WT datasets in the GEO database to identify hub genes involved in WT development. The hub genes were all found to be up-regulated and might serve as new biomarkers for predicting the prognosis of WT. Survival analysis was used to predict the prognosis of these hub genes. *KIF2C* was the gene most closely associated with WT survival. We found that *KIF2C* was up-regulated in WT patients. There is a considerable correlation between the high expression of *KIF2C* and the poor OS of WT patients. The expression of *KIF2C* in WT was further verified by qRT-PCR and IHC. The expression of *KIF2C* may be associated with regulating mitosis and cell cycle. Further investigation is needed to explore these mechanisms. The biological behavior, molecular mechanism and the role of *KIF2C* in WT immune cell infiltration need to be further examined and characterized to obtain more accurate correlation results. Further analysis will be carried out to determine which specific immune cells play a significant role in the WT tumor microenvironment and which functions these infiltrating immune cells carry out in order to better understand the relationship between high expression of *KIF2C* and poor prognosis in WT patients. This study may provide new insights into the diagnosis and effective therapeutic strategies for WT in the future.

### Supplementary Information

Supplementary Information 1.

Supplementary Information 2.

## Data Availability

The datasets supporting the conclusions of this article are available in the GEO database (http://www.ncbi.nlm.nih.gov/geo/). All data generated or analyzed during this study are included in the article.
