# Supplementary material for: Bioinformatical analysis of the key differentially expressed genes for screening potential biomarkers in Wilms tumor
Source: Sci Rep. 2023 Sep 16;13:15404. doi: 10.1038/s41598-023-42730-w (PMC10505208; doi:10.1038/s41598-023-42730-w)
Supplement: Supplementary file 1 — Supplementary Information 1. [file 41598_2023_42730_MOESM1_ESM.pdf]

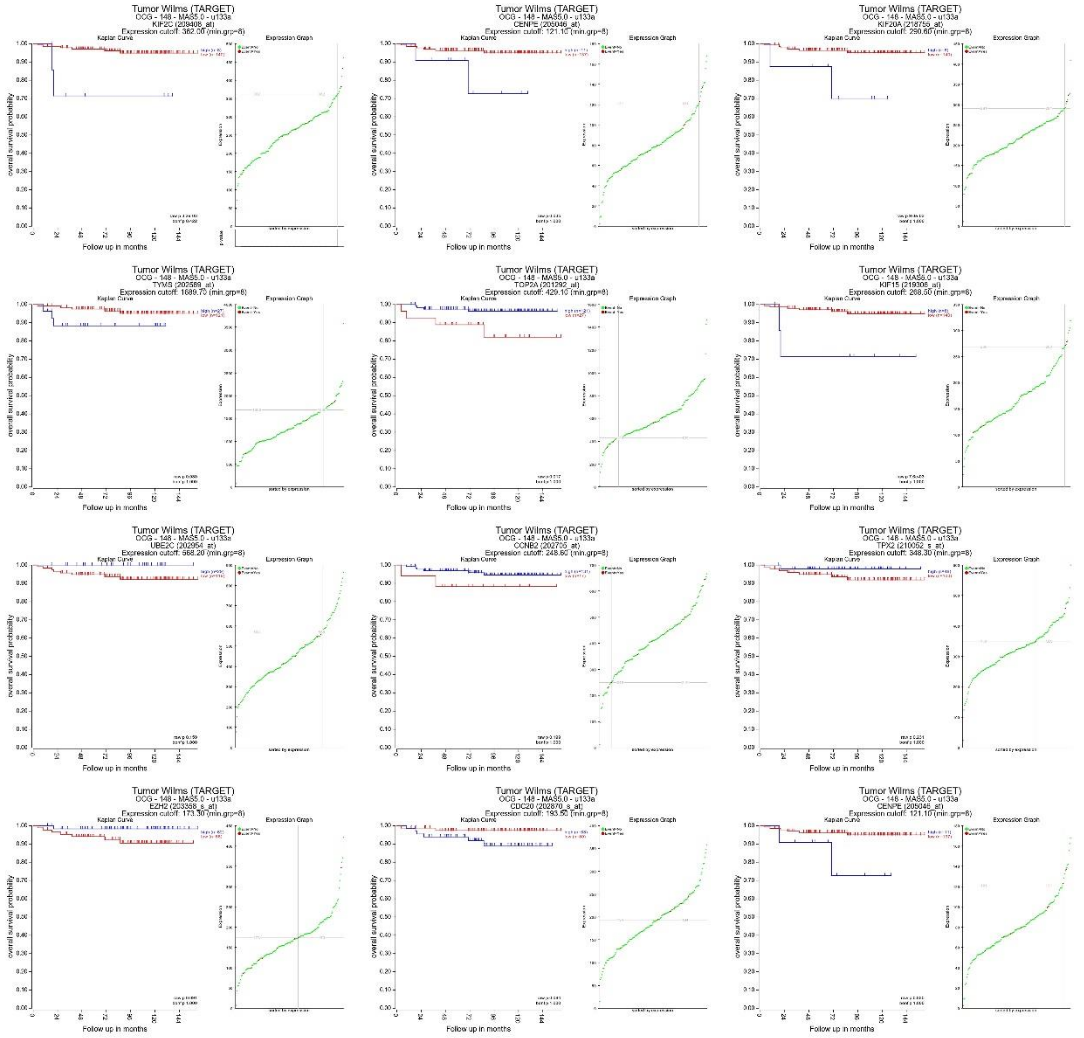

Supplemental figure 1. The relationship between 12 hub genes expression and the overall survival of patients with Wilms tumor. *KIF2C*, kinesin family member 2C; *CENPE*, centromere protein E; *KIF20A*, kinesin family member 20A; *TYMS*, thymidylate synthetase; *TOP2A*, topoisomerase II alpha; *KIF15*, kinesin family member 15; *UBE2C*, ubiquitin-conjugating enzyme E2C; *CCNB2*, cyclin B2; TPX2, TPX2 Microtubule Nucleation Factor; *EZH2*, enhancer of zeste 2 polycomb repressive complex 2 subunit; *CDC20*, cell division cycle 20; CENPF, centromere protein F.
